# Study on construction mechanic characteristics and construction optimization of super-large cross-section tunnel portal under shallow buried depth and asymmetrical loading: A case study in Southwest China

**DOI:** 10.1371/journal.pone.0316736

**Published:** 2025-01-03

**Authors:** Hao Chen, Haozan Zheng, Bolong Liu, Man Huang, Jiawei Liu

**Affiliations:** 1 School of Civil Engineering, Shaoxing University, Shaoxing, China; 2 Key Laboratory of Rock Mechanics and Geohazards of Zhejiang Province, Shaoxing University, Shaoxing, China; 3 School of Urban Rail Transportation, Soochow University, Suzhou, China; 4 Zhejiang Natural Resources Group Spatial Information Co., Ltd, Hangzhou, China; Faculty of Engineering, University of Rijeka, CROATIA

## Abstract

The excavation of the super-large cross-section tunnel portal section is prone to causing serious engineering distresses. The key factors to ensure the safe construction of portal section are to clarify the construction mechanic characteristics and select a reasonable construction scheme. In this paper, a bidirectional six-lane expressway tunnel in Southwest China was selected as an engineering case. Three excavation schemes, namely, the three-bench seven-step excavation method (TEM), the central diaphragm method (CDM), and the double side drift method (DSDM), were compared and analyzed. Findings revealed that due to the effect of the asymmetrically loaded and super-large cross-section, the surrounding rock deformation and supporting structure stress at the deep buried side were greater than those at the shallow buried side. The CDM and DSDM could reduce the tunnel span and provide temporary support in time, which could effectively control the surrounding rock deformation and improve the structural stress and the slope stability. According to the topographic condition, the excavation sequence of the DSDM was optimized. Excavating the shallow buried side drift first could alleviate the surrounding rock deformation and improve the slope stability in the early stage of construction. Finally, the optimal excavation scheme was successfully implemented.

## 1 Introduction

In the face of the expansion of urban scale and the rise of the logistics industry, numerous large cross-section or super-large cross-section tunnels have been placed into construction in China to improve the highway transportation capacity and alleviate the congestion caused by the continuous growth of the number of vehicles. At present, no uniform standard exists for the division of super-large cross-section tunnels. The International Tunnel Association stipulates that a tunnel with a net cross-section area greater than 100 m^2^ is a super-large cross-section tunnel, whereas the Japan Tunnel Association defines it as an excavated cross-section area exceeding 140 m^2^ [1]. In the “Code for design of road tunnel (JTG 3370.1–2018)”, a tunnel with a span of more than 18 m is classified as an extra-large-span tunnel [2]. Compared with conventional cross-section or span tunnels, super-large cross-section tunnels have large excavation area, lower high-span ratio, and complicated construction procedures. The following problems exist in the construction of super-large cross-section tunnels: ① The stress redistribution of tunnel becomes unfavorable after excavation. ② Substantial stress is concentrated at the arch foot, and the foundation bearing capacity at the tunnel site must be high. ③ After excavation, the surrounding rocks produce large, loose ground pressure, which leads to the instability of the tunnel arch. ④ The supporting structure has limited functionality and capacity [3–7]. Combined with large-scale model experiment and numerical simulation, Sun et al. [8] studied the mechanical response law of surrounding rock during the construction of super-large-span and variable-section tunnels, and revealed the deformation and stress release mechanism of the surrounding rock. Ji et al. [9] compared the cumulative damage effects of the surrounding rock during each excavation stage of a large section tunnel using an established rock damage model and equivalent simulation method, and then proposed a safety evaluation standard for the surrounding rock. Zhong et al. [10] independently developed a large-scale 3D test platform to realize the disaster process of water and mud inrush in large section tunnels crossing the water-rich fault fracture zone, explored the mechanism of disaster occurrence, and proposed the optimal parameters for curtain grouting reinforcement. Jiang et al. [11] analyzed the ground subsidence law and influencing factors during the excavation of large section shallow buried tunnels under various compression states with actual engineering site investigation and simulation methods, and quantitatively described the relationship between ground subsidence and tunnel excavation area. Zheng et al. [12] established a refined 3D finite element model of ultra-large diameter shield tunnels, deeply analyzed the construction disturbance response and the mechanism of segment instability, and then proposed the corresponding reinforcement measures.

Affected by topography and geomorphology, the shallow buried and biased stratum is the most common adverse geological condition during the tunnel portal construction. Under the influence of shallow buried and biased stratum, the tunnel portal structure bears uneven loose load, which is prone to evident stress concentration and excessive deformation on the supporting structure that result in cracking, spalling, and failure of the concrete, surface cracks, supporting structure failure, and even tunnel collapse [13–17]. In addition, due to the shallow buried depth of the portal, the abundant local precipitation and the occurrence of surface cracks during the construction could degrade the mechanical properties of the surrounding rock and further increase the excavation difficulty of the tunnel portal [18–23]. When building a super-large cross-section tunnel in shallow buried and asymmetrical stratum, if the construction mechanic characteristics of the structure are not well understood or the excavation schemes adopted are unreasonable, this situation would easily cause numerous construction distresses and threaten the life safety of construction personnel.

Selecting a scientific, reasonable excavation scheme is the key factor to ensure the smooth construction of super-large cross-section tunnels in a complex stratum. To control the surrounding rock deformation and improve the tunnel face stability, the sequential excavation method (SEM) is generally used to divide the super-large cross-section tunnel into several temporary drifts in the horizontal or vertical direction for construction [24]. Among these SEMs, the Three-Bench Seven-Step Excavation Method (TEM), the Central Diaphragm Method (CDM), and the Double Side Drift Method (DSDM) are widely used in tunnel construction due to their unique advantages.

Due to the advantages such as high efficiency (the large space is conducive to mechanization), strong adaptability (suitable for various excavation span and section types), satisfactory engineering economy and high safety (full utilization of core soil), as an improvement of the Three-Bench method, the TEM has been widely used in the excavation of large cross-section loess tunnels [25]. Zhao et al. [26] systematically summarized the existing main problems and key technologies of the design and construction of large cross-section loess tunnels in China, and optimized the construction parameters in the TEM such as excavation footage, bench height, and the distance between the benches through the field test. By summarizing and analyzing a large amount of field monitoring data of large span loess tunnel excavated by the TEM, Luo et al. [27] found that the deformation of initial concrete could be divided into three stages: rapid growth, continuous growth and gradual stabilization. The CDM has always been one of the main excavation methods for shallow buried and asymmetrically loaded tunnel portal because of its advantages, such as short closing time of each drift, uniform stress on support structure, and high support stiffness of middle partition wall [28]. Li et al. [29] and Chen et al. [30] improved the traditional CDM to improve the service efficiency of mechanical equipment and reduce the excavation cycle time and construction cost. The main difference was that the lower bench was divided into three parts and the installation of temporary support for lower bench was eliminated, which had better control effect on invert uplift. The DSDM is more effective in controlling the surrounding rock deformation and surface subsidence, and it is more suitable for the excavation of super-large cross-section tunnels in a complex stratum [24]. To address the problem of excessive surface subsidence during the excavation of shallow buried large-span tunnels when crossing the existing expressway in Shenzhen, Cao et al. [31] optimized the DSDM and the auxiliary measures, including timely closing the initial support, accelerating the construction of the secondary lining, and shortening the distance between the secondary lining and the tunnel face. The surface subsidence and surrounding rock deformation were considerably decreased after the optimization measures were adopted. Zhao et al. [32] took the Xi’an Metro Line 4 as a case and considered that the DSDM had excellent economic benefits and played an evident control effect on surface subsidence, vault settlement, and horizontal convergence of a large cross-section metro tunnel in a shallow loess stratum.

As the throat section of the entire tunnel project, the portal has always been the difficulty and focus of construction. When multiple unfavorable factors such as shallow buried depth, asymmetrical loading, broken surrounding rock, and super-large cross-section are coupled, the difficulty of constructing the tunnel portal is remarkably increased. However, scholars have seldom researched the construction mechanical characteristics and excavation scheme optimization of the tunnel portal under the coupling of multiple unfavorable factors. In this paper, a super-large cross-section tunnel in Southwest China was taken as a case. With the help of numerical simulation and field monitoring methods, the surface subsidence, surrounding rock deformation, support stress, and slope stability of the tunnel portal during the construction of the TEM, CDM, and DSDM were compared and analyzed. Finally, a set of scientific, reasonable excavation schemes was obtained and applied to actual engineering successfully. The research results can provide valuable reference and basis for similar projects in the future.

## 2 Project overview

### 2.1 Engineering geological conditions

**Fig 1:**
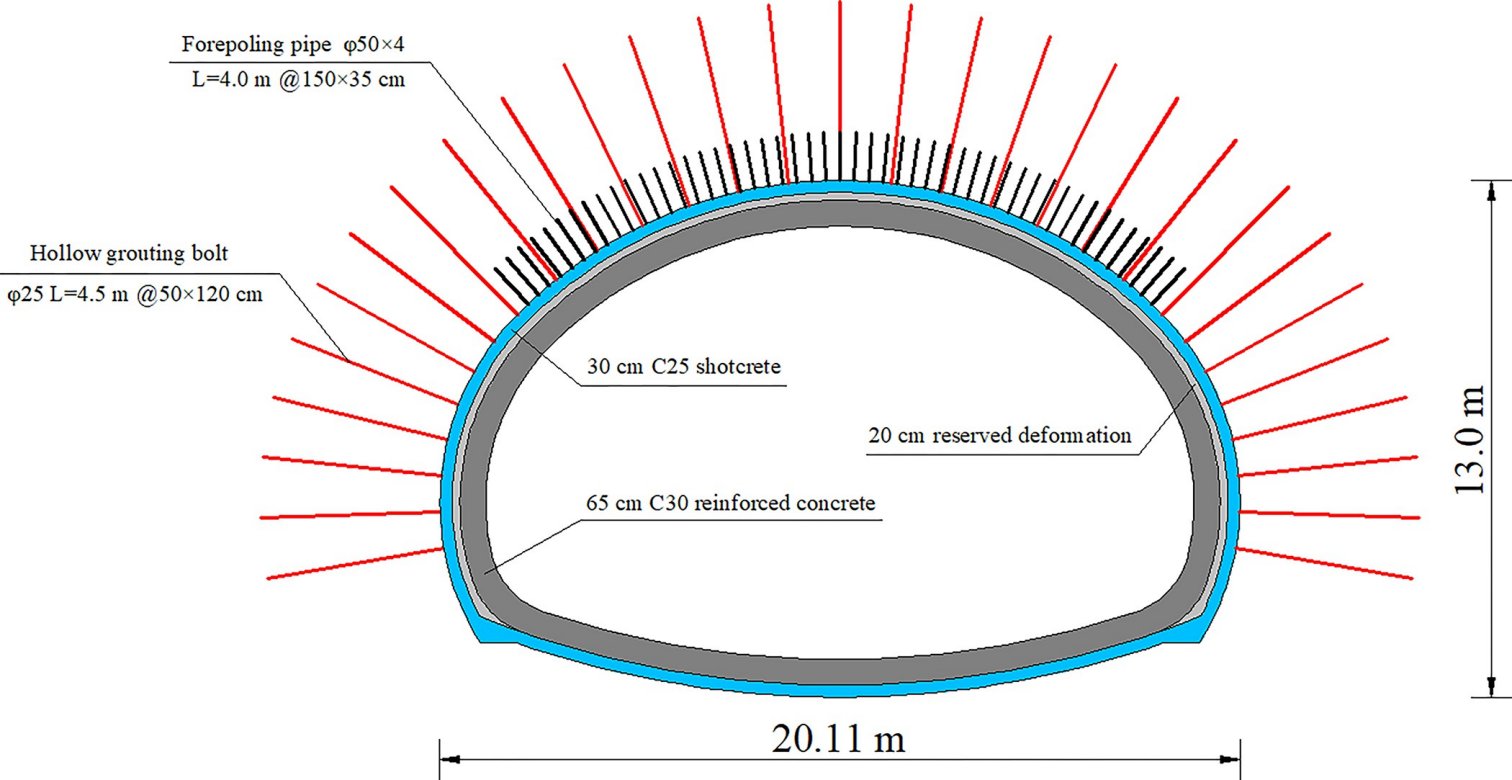
Cross section and supporting structure of tunnel. The tunnel is in the karst area and belongs to low and middle mountain landform with large topographic fluctuation. The surrounding rocks of the tunnel are mainly yellow-brown Pleistocene Diluvial clay and gravel, with a layer thickness of 0.7–15.6 m, and light gray Cambrian Upper Middle Order Loushanguan Group dolomite limestone and dolomite, with a thickness of 28.7–38.8 m. The left tunnel portal is on a slope. The slope is asymmetrical in topography, with a slope gradient of about 25°, which is thick on the left while thin on the right. Therefore, a typical topographic asymmetrical loading phenomenon is in the left tunnel portal. The minimum buried depth of the left tunnel portal is about 5.0 m. The right tunnel portal is symmetrical in topography, and the slope is stable. The minimum buried depth of the right tunnel portal is about 5.6 m.

The studied tunnel is a separated bidirectional six-lane expressway tunnel with the same width as roadbed. The maximum excavation span of the tunnel reaches 20.11 m, whereas the maximum excavation height reaches 13.0 m (Fig 1). The high-span ratio of the tunnel is 0.646, which is equivalent to that of most single-hole four-lane tunnels. The excavation area of the tunnel exceeds 200 m^2^. Whether referring to the recommended standards of the International Tunnel Association, the Japan Tunnel Association, or the “Code for design of road tunnel (JTG 3370.1–2018)” [1,2], the studied tunnel belongs to a super-large cross-section tunnel or an extra-large-span tunnel.

The surface water in the tunnel site is insufficient, and no surface water source is available. The groundwater is mainly fissure water and karst water in rock mass. The karst is developed in limestone and dolomitic limestone, and has satisfactory connectivity and minimal water. The groundwater is mainly supplied by atmospheric precipitation, and the groundwater level is about 12.5–28.0 m below the tunnel invert.

The studied tunnel was constructed by using the new Austrian method, and the main support measures are shown in Fig 1. The grouting small pipes adopted to provide the advanced support had a diameter of 50 mm, a length of 4.0 m, and a circumferential spacing of 35 cm, which were arranged within 120° of the tunnel arch. The primary support included 4.5 m long hollow grouting anchor bolt with 50 cm circumferential spacing and 120 cm longitudinal spacing, I25b steel arch with longitudinal spacing of 50 cm, and *φ*8 reinforcement mesh 20 × 20 cm double layer laid; 30 cm thick C25 concrete was sprayed. The 65 cm thick reinforced concrete was adopted as the secondary lining, and a reserved deformation of 20 cm was between the shotcrete layer and the secondary lining layer.

### 2.2 Excavation schemes

Due to the shallow buried depth, asymmetrical loading in topography, and super-large excavation area, a reasonable excavation scheme must be adopted to ensure the safe, smooth construction of the left tunnel portal. According to the existing construction conditions, three excavation methods were proposed, namely, the TEM, the CDM, and the DSDM. The three methods are introduced as follows.

**Fig 2:**
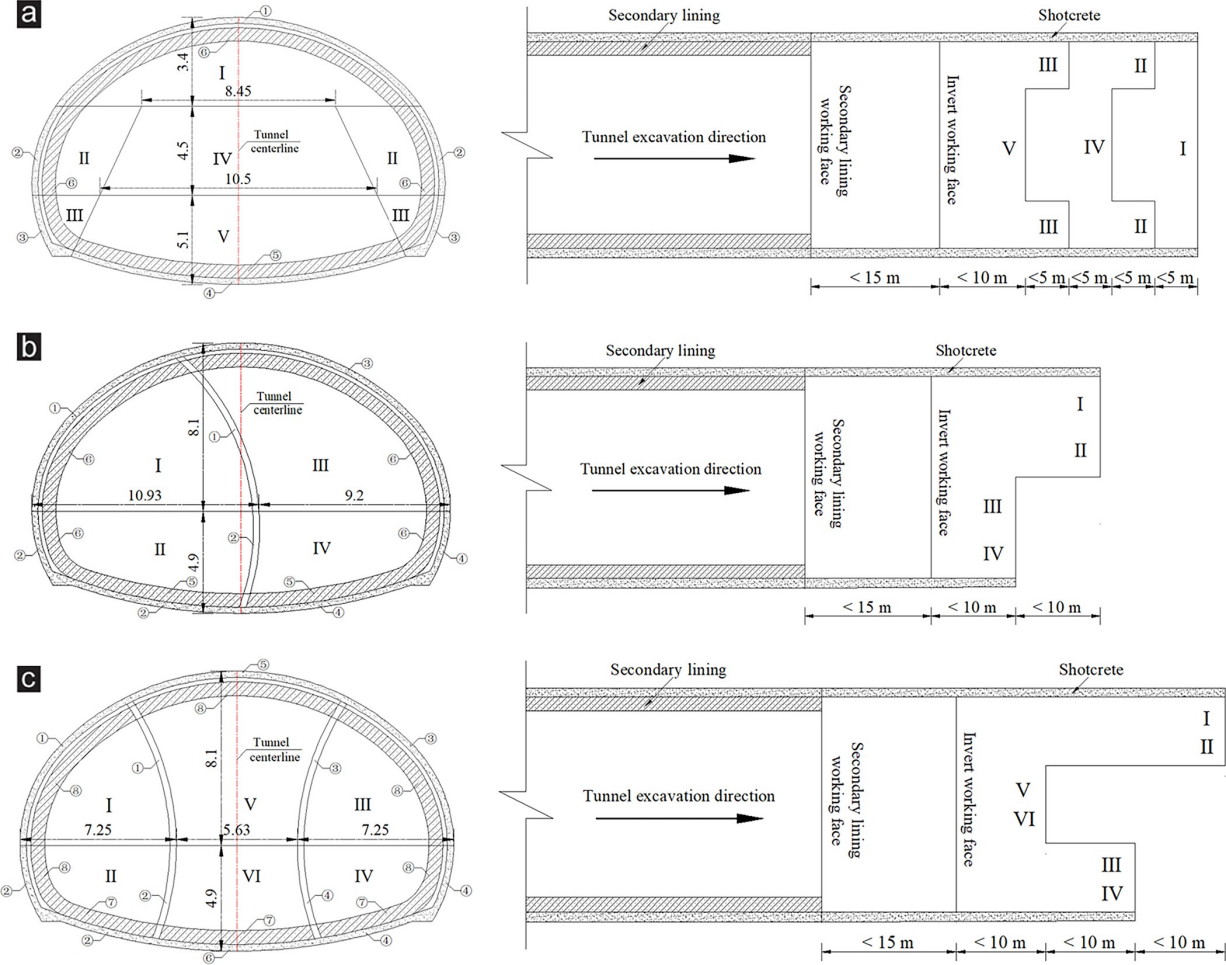
Excavation sequences of each excavation method (unit: m). (a) TEM, (b) CDM, (c) DSDM.

Fig 2(A) illustrates the specific excavation sequence of the TEM: (1) The upper bench Ⅰ is excavated, the excavation step length is controlled within 1.0 m each time (the step length of other drifts or benches is controlled within 1.0 m each time), and then the corresponding lengths of the primary support ① is implemented. (2) The left and right drifts Ⅱ of the middle bench are excavated, and then the corresponding lengths of the primary support ② is implemented. (3) The left and right drifts Ⅲ of the lower bench are excavated, and then the corresponding lengths of the primary support ③ is implemented. (4) The core soil Ⅳ of the middle bench is excavated. (5) The core soil Ⅴ of the lower bench is excavated, and then the corresponding lengths of the primary support ④ is implemented. (6) The invert ⑤ is poured and backfilled. (7) The secondary lining ⑥ is implemented. During the excavation, the excavation distance between adjacent drifts or benches should be controlled within 5 m.

Fig 2B shows the specific excavation sequence of the CDM: (1) The left upper drift Ⅰ is excavated, the excavation step length is controlled within 1.0 m each time (the step length of other drifts is controlled within 1.0 m each time), and then the corresponding lengths of the primary support and temporary support ① are implemented. (2) The left lower drift Ⅱ is excavated, and then the corresponding lengths of the primary support and temporary support ② are implemented. (3)The right upper drift Ⅲ is excavated, and then the corresponding lengths of the primary support ③ is implemented. (4) The right lower drift Ⅳ is excavated, and then the corresponding lengths of the primary support ④ is implemented. (5) The invert ⑤ is poured and backfilled. (6) The temporary support is removed, and the secondary lining ⑥ is implemented. During the excavation, the excavation distance between adjacent drifts should be controlled within 5 m.

Fig 2C exhibits the specific excavation sequence of the DSDM: (1) The left upper drift Ⅰ is excavated, the excavation step length is controlled within 1.0 m each time (the step length of other drifts is controlled within 1.0 m each time), and then the corresponding lengths of the primary support and temporary support ① are implemented. (2) The left lower drift Ⅱ is excavated, and then the corresponding lengths of the primary support and temporary support ② are implemented. (3) The right upper drift Ⅲ is excavated, and then the corresponding lengths of the primary support and temporary support ③ are implemented. (4) The right lower drift Ⅳ is excavated, and then the corresponding lengths of the primary support and temporary support ④ are implemented. (5) The middle upper drift Ⅴ is excavated, and then the corresponding lengths of the primary support ⑤ is implemented. (6) The middle lower drift Ⅵ is excavated, and then the corresponding lengths of the primary support ⑥ is implemented. (7) The invert ⑦ is poured and backfilled. (8) The temporary support is removed, and the secondary lining ⑧ is implemented. During the excavation, the excavation distance between adjacent drifts should be controlled within 5 m.

### 3 Establishment of numerical model

Previous studies indicated that the excavation methods had a substantial influence on the deformation characteristics of the surrounding rock and the stress distribution of the supporting structure. Numerical simulation has been widely used in the decision making of excavation methods due to its low-cost controllability and repeatability [33]. In this paper, FLAC3D software was used to compare and analyze the surrounding rock deformation, the supporting structure stress, and the slope stability of tunnel portal under various excavation methods.

### 3.1 Model size and boundary conditions

**Fig 3:**
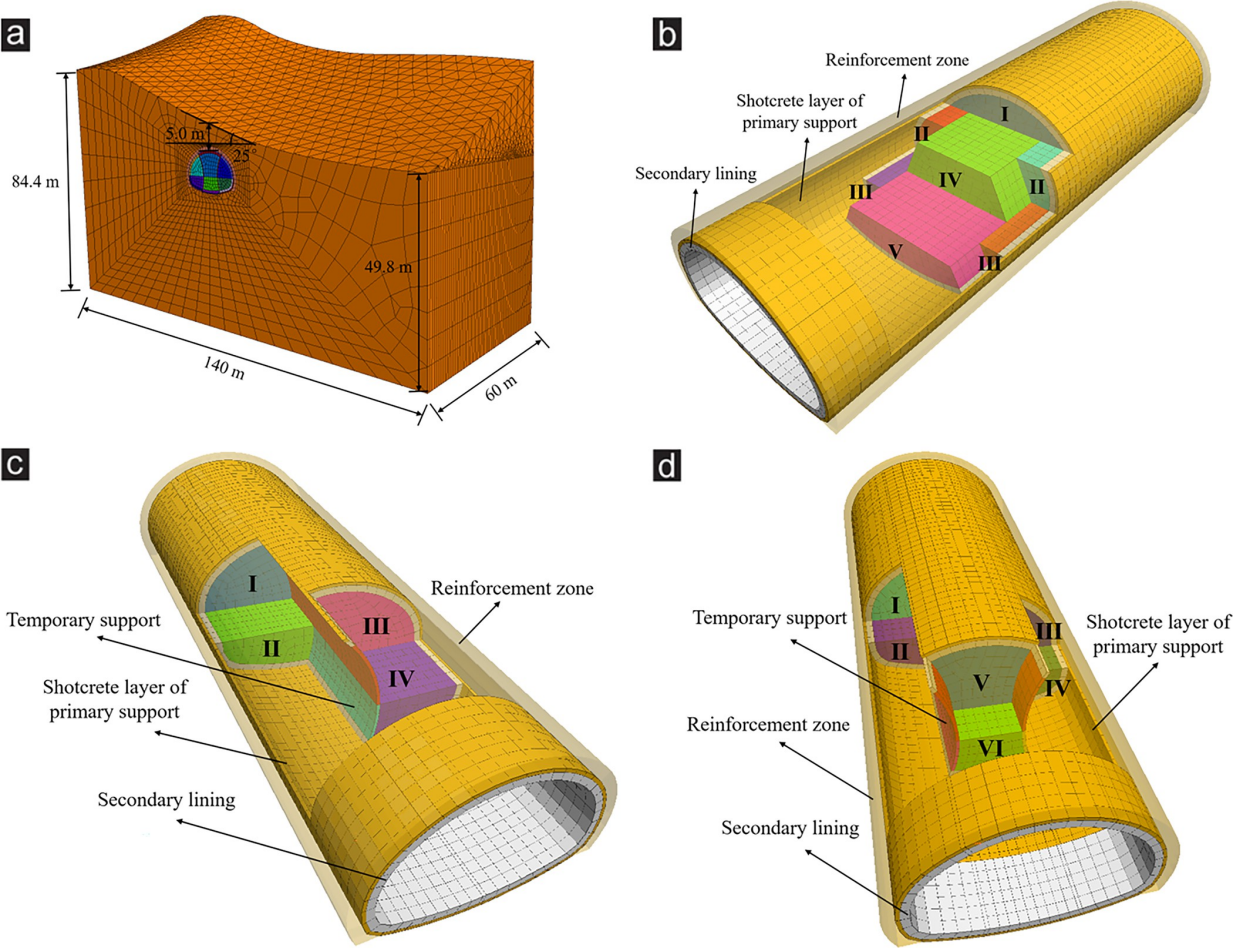
Numerical model. (a) FD mesh of the model, (b) TEM, (c) CDM, (d) DSDM.

The left tunnel portal section (ZK119+575-ZK119+635) with serious shallow buried depth and asymmetrical loading was selected as the study object. The 3D numerical model was established according to the geological and topographic conditions of the tunnel portal, as shown in Fig 3. Fig 3A presents the overall tunnel model. To eliminate the boundary effect, the model boundaries were set three times the tunnel width in the horizontal direction, and the total width was 140 m. In the vertical direction, the distance between the model bottom and the tunnel arch bottom was three times the tunnel height. The upper boundary was taken to the surface. The topography of the slope at the tunnel portal was thick on the left and thin on the right. The slope gradient was about 25°, and the minimum buried depth of the tunnel was about 5.0 m. The longitudinal length of the model was 60 m. Because the simulated tunnel portal was in the shallow buried section, no tectonic stress was applied in the simulation. The bottom of the model was fixed. The normal displacement of four vertical boundaries was restricted, and the top of the model was a free boundary. Fig 3B–3D show the specific excavation sequence simulation of the TEM, CDM and DSDM, respectively.

### 3.2 Model parameters

In the simulation, the surrounding rock was a continuous homogeneous and isotropic elastic–plastic material, and it followed the Mohr–Coulomb criterion. The surrounding rocks at the tunnel portal were mainly strongly weathered dolomitic limestone and a small amount of clay and gravel. According to the preliminary geological survey data, no karst landforms such as caves were in the tunnel portal, so the effect of caves on tunnel portal construction was not considered in the simulation. According to the laboratory test results and geological survey reports, the physical and mechanical parameters of the surrounding rock in the simulation are listed in Table 1. The grouting reinforcement effect of the advanced small pipes and anchor bolts was achieved by improving the cohesion and internal friction angle of the surrounding rock to form a reinforcement area [28,33]. The elastic element was used to simulate the primary support, temporary support, and secondary lining, and the “Cable” element was used to simulate the system bolts.

**Table 1:** Parameters of rock and supporting structures used in simulation.

Materials	Unit weight (kN/m^3^)		Elastic modulus (MPa)	Poisson’s ratio		Cohesion (kPa)	Internal friction angle (°)
Strongly weathered dolomitic limestone	19.8	1500		0.38	150		24
Reinforced area	19.8	1650		0.38	165		25.2
Primary support	25	28800		0.2	—		—
Secondary lining	25	31500		0.2	—		—
Temporary support	22	25200		0.25	—		—
System bolt	78.5	210000		0.3	—		—

### 3.3 Verification of numerical model

**Fig 4:**
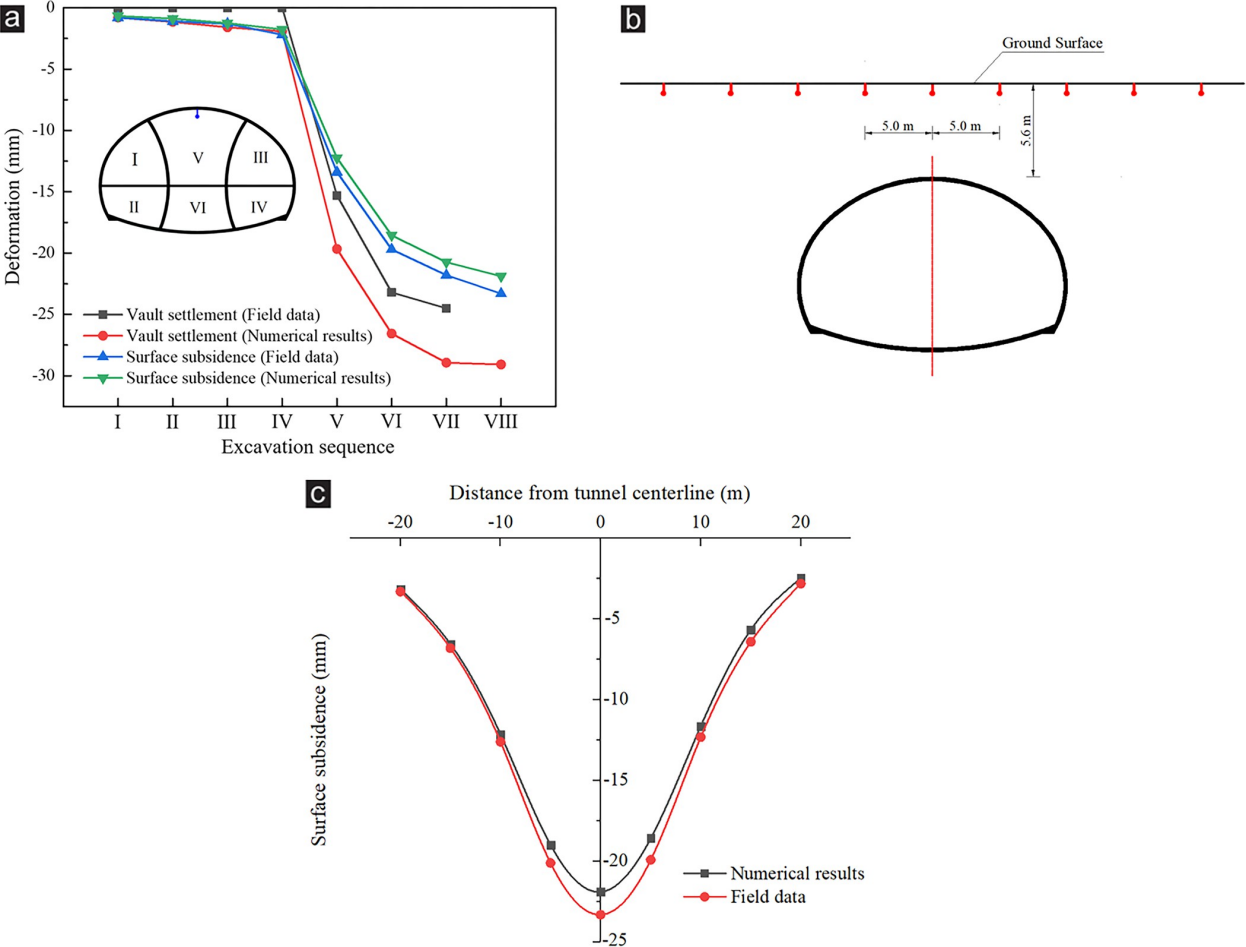
Comparison of surrounding rock deformation between numerical simulation and field monitoring at YK119+595 section of right tunnel. (a) Surface subsidence and vault settlement under each excavation sequence, (b) Monitoring point of the surface subsidence, (c) Surface subsidence. The DSDM was adopted to excavate the right tunnel portal, and the excavation sequence is shown in Fig 4a. The vault settlement monitoring points had not been installed during steps Ⅰ–Ⅳ, so the field data were 0. After pouring the lining concrete, the vault settlement monitoring points were covered up, so no field data were in step Ⅷ. After removing the temporary support (step Ⅶ), the measured value of the vault settlement was 24.5 mm, which was less than the simulation result of 28.93 mm. During the actual tunnel excavation, the displacement of a certain section of the tunnel could be divided into four stages, namely, advanced deformation, deformation due to stress release, primary support deformation, and secondary lining deformation. Due to limitations of conventional monitoring techniques and instruments, the field monitoring of the tunnel displacement was performed after excavation, and only the primary support deformation and secondary lining deformation could be monitored [34], whereas the entire process of tunnel displacement could be monitored with the aid of numerical simulation. Therefore, the displacement value of the simulation results would be greater than the field monitoring value. Compared with tunnel deformation monitoring, the surface subsidence could be monitored in real time, so the monitoring data agreed with the simulation results. Fig 4b presents the layout of the surface subsidence monitoring points. Fig 4a shows that under each excavation sequence, the deformation trend of simulated settlement value was consistent with the field data. In Fig 4c, due to the absence of asymmetrical loading in the right tunnel portal, the simulated and measured values of surface subsidence presented a symmetrical distribution, and their distribution trends were also relatively consistent. These all indicated that the established numerical model and the selected material parameters were reliable and reasonable.

After the numerical model was established, the field monitoring data or laboratory test results were used to verify the rationality of the numerical model and material parameters. During the actual construction, the topography of the right tunnel portal was symmetrical, and the construction difficulty was less than that of the left tunnel portal, so it was excavated as the first tunnel. The settlement monitoring data at the YK119+595 section of right tunnel portal were selected to verify the numerical model and parameters. The comparison between the simulation results and field monitoring data is shown in Fig 4.

## 4 Analysis and discussion of simulation results

### 4.1 Deformation analysis of surrounding rock

**Fig 5:**
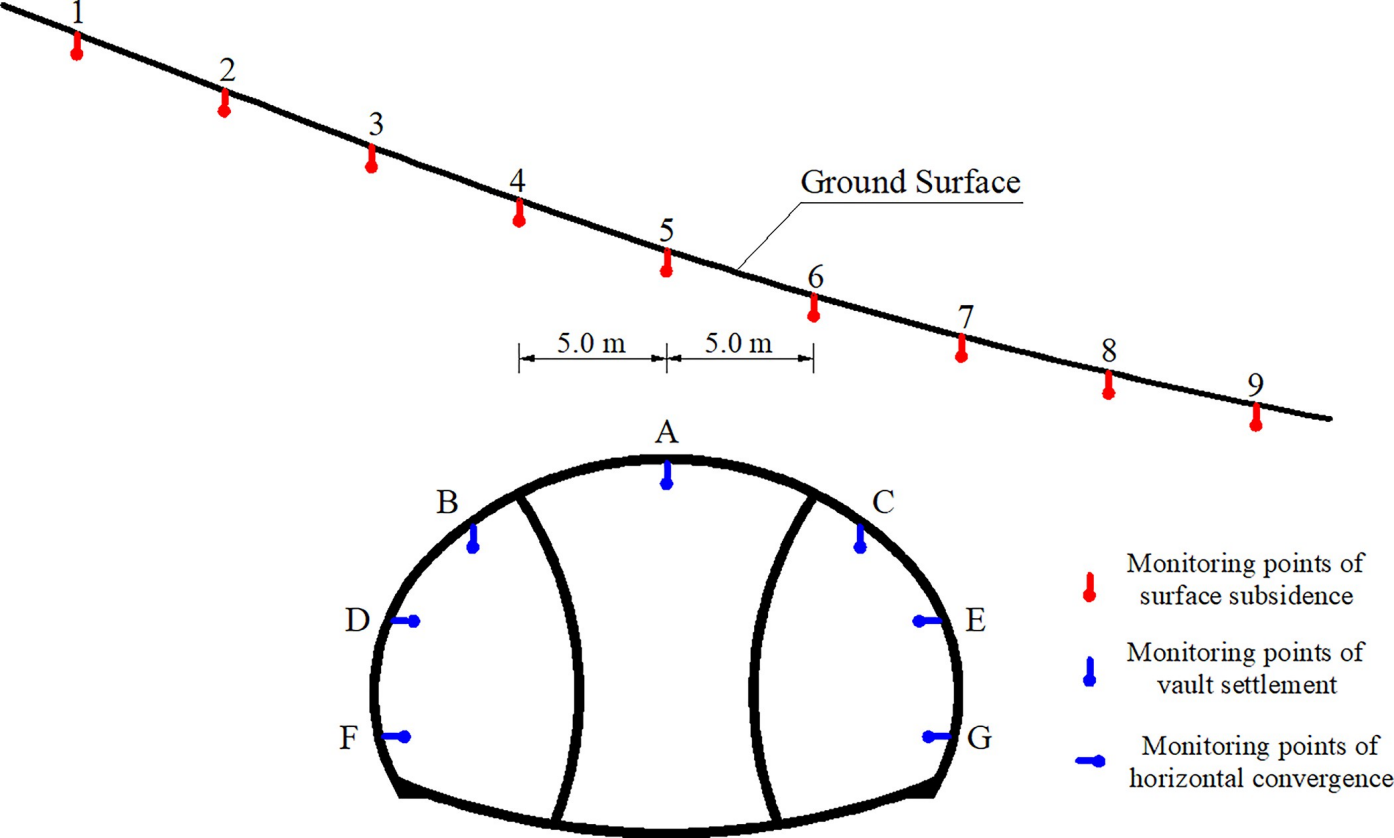
Distribution of the monitoring points.

Fig 5 shows the specific layout of field monitoring points for surface subsidence and surrounding rock deformation in the left tunnel portal during actual construction. Nine points were arranged on the slope surface, and the horizontal distance between adjacent points was 5.0 m, of which the Number 5 point was directly above the tunnel axis.

**Fig 6:**
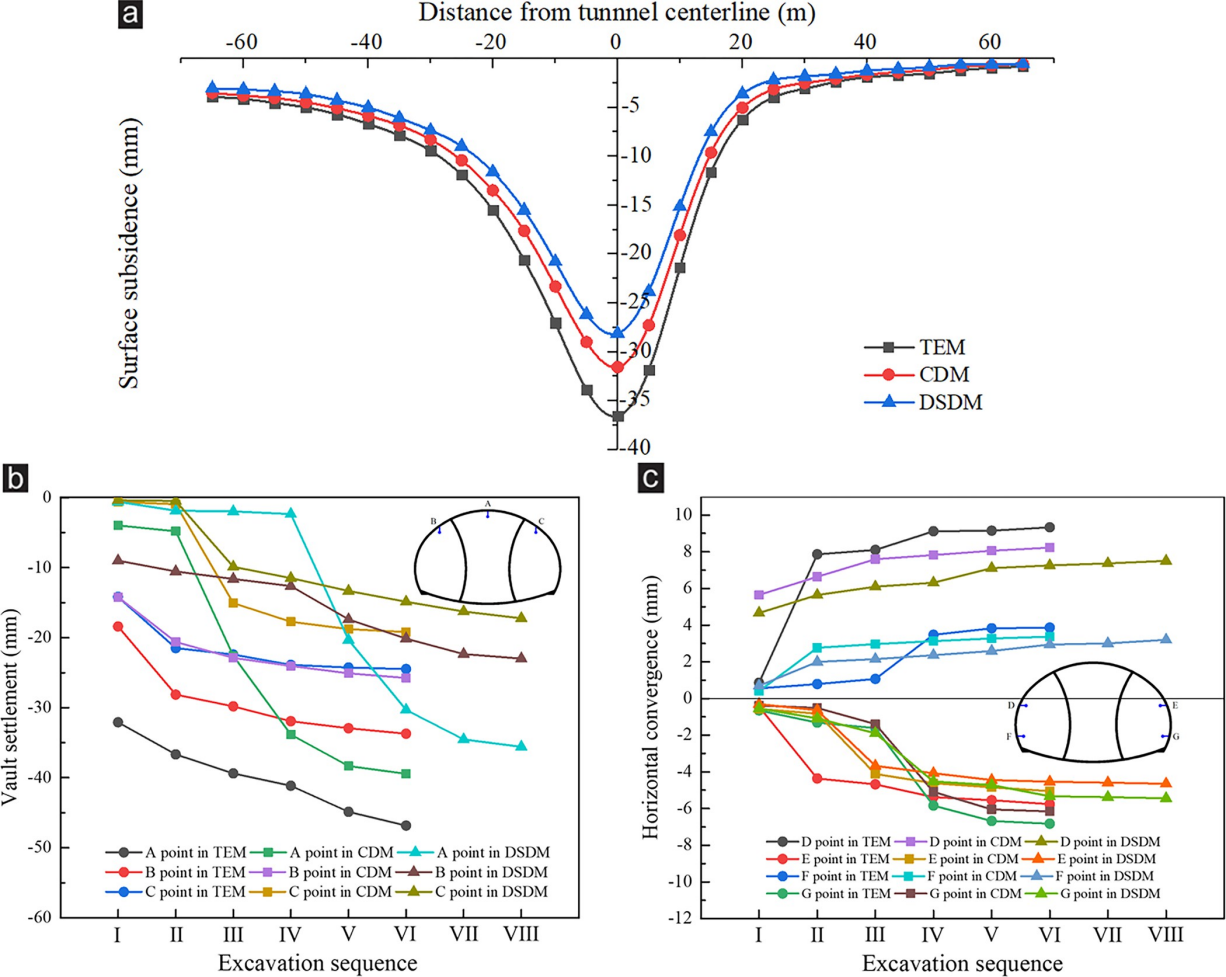
Surrounding rock deformation at ZK119+580 section under various excavation methods. (a) Surface subsidence, (b) Vault settlement, (c) Horizontal convergence. In Fig 6b and 6c, the abscissa axis represents the excavation sequence of each method, as shown in Fig 2. Step Ⅵ in the TEM was to implement the overall secondary lining. Steps Ⅴ and Ⅵ in the CDM and steps Ⅶ and Ⅷ in the DSDM corresponded to the removal of temporary support and the construction of secondary lining, respectively. The surrounding rock deformation produced by the TEM was the most substantial, followed by that of the CDM, and that of the DSDM was the smallest. The maximum vault settlement appeared at point A (the tunnel vault), which reached 46.85, 39.44 and 35.58 mm. The maximum peripheral convergence appeared at point D (the left arch waist), which reached 9.55, 8.23, and 7.50 mm. Under the influence of topographic bias, the surrounding rock deformation at the deep buried side was considerably greater than that at the shallow buried side. For instance, in the TEM, the settlement value at point B was 9.22 mm larger than that at point C, which was about 27.35%.

Fig 6A–6C present the simulation results of surface subsidence, vault settlement, and horizontal convergence at the ZK119+580 section of the left tunnel portal under the three excavation methods. Fig 6A shows that after the tunnel was excavated, substantial subsidence occurred on the slope, and the maximum subsidence was directly above the tunnel axis (No. 5). Affected by the topographic bias, the subsidence presented an evident asymmetry. The overall characteristic was that the subsidence at the deep buried side was considerably greater than that at the shallow buried side. Fig 6A reveals that the subsidence value was affected by excavation method. The largest surface subsidence was caused by the TEM that reached 36.58 mm, the CDM that reached 31.68 mm, and the DSDM that reached 28.12 mm.

### 4.2 Stress analysis of primary support concrete

**Fig 7:**
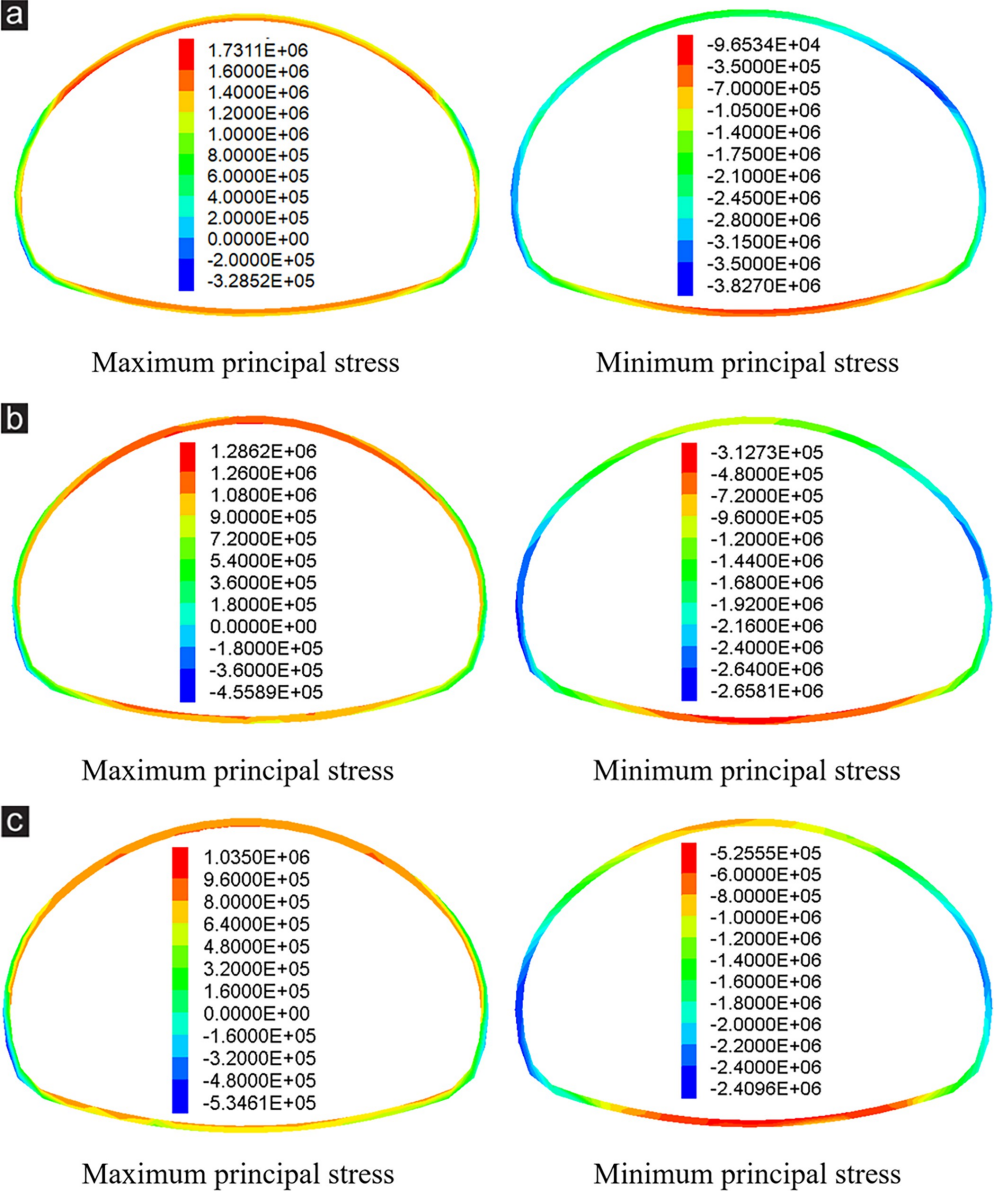
Stress of primary support concrete at the ZK119+580 section under various excavation method (Unit: Pa). (a) TEM, (b) CDM, (c) DSDM.

Previous studies indicated that cracking and spalling of primary concrete is a common engineering distress during the construction of a tunnel portal under shallow buried depth and asymmetrical loading [21,28], so its cracking mechanism must be analyzed. The principal stresses of the primary support concrete at the ZK119+580 section of the left tunnel portal under various excavation methods are listed in Fig 7. When the primary support concrete was closed into a ring, the vault and invert were subjected to tensile stress, and the tensile stress on the vault was more serious than that on the invert. Comparing the stress value under the three excavation methods reveals that the tensile stress at vault was the most serious after excavation by the TEM and reached 1.731 MPa (Fig 7A), which was close to the ultimate tensile strength of C25 concrete [2,17]. The tensile stress at vault reached 1.286 and 1.035 MPa after excavation by the CDM and DSDM (Fig 7B and 7C), respectively, which had a certain safety reserve.

Affected by the biased stratum, the minimum principal stress distribution on the primary support concrete presented a substantial asymmetry, and the stress at the deep buried side was more serious than that at the shallow buried side. After excavation by the TEM, the maximum compressive stress appeared at the left arch waist and right arch shoulder, and reached 3.827 MPa (Fig 7a). After excavation by the CDM and DSDM, the maximum compressive stress appeared at the left arch waist and the right arch waist, and reached 2.658 and 2.409 MPa (Fig 7B and 7C), respectively. The stress at the left arch waist was more serious than that on the right. However, no matter which excavation method was adopted, the compressive stress on the primary support concrete was much less than the ultimate compressive strength of C25 concrete [2,17], which had a high safety reserve.

### 4.3 Analysis of slope stability

Slope cracking and instability often occur when building a tunnel portal in shallow buried and biased strata [21,28]. Thus, slope stability is also a key factor to be considered in the construction of tunnel portal. The calculation of slope safety factor (*FS*) based on strength reduction method was adopted in this paper.

The strength reduction method has been widely applied in geotechnical materials using the Mohr–Coulomb failure criterion, and the shear strength in the Mohr–Coulomb geotechnical materials refers to the cohesive force *c* and internal angle *φ*. The process of strength reduction is to continuously reduce (when the geotechnical material is initially in a stable state) or continuously increase (when the geotechnical material is initially in an unstable state) the cohesive force *c* and internal angle *φ* of the geotechnical material until the material fails. The specific formula is as follows:

$$\left\{ \begin{array}{l} c_{F}=c/FS\\ \varphi_{F}=\tan^{-1}(\tan\varphi /FS) \end{array}\right.$$ (1)

In the formula, *c*, *φ*, *c*_*F*_ and *φ*_*F*_ are the cohesive force and internal friction angle of the geotechnical materials before and after reduction, respectively, and *FS* is the safety factor.

**Fig 8:**
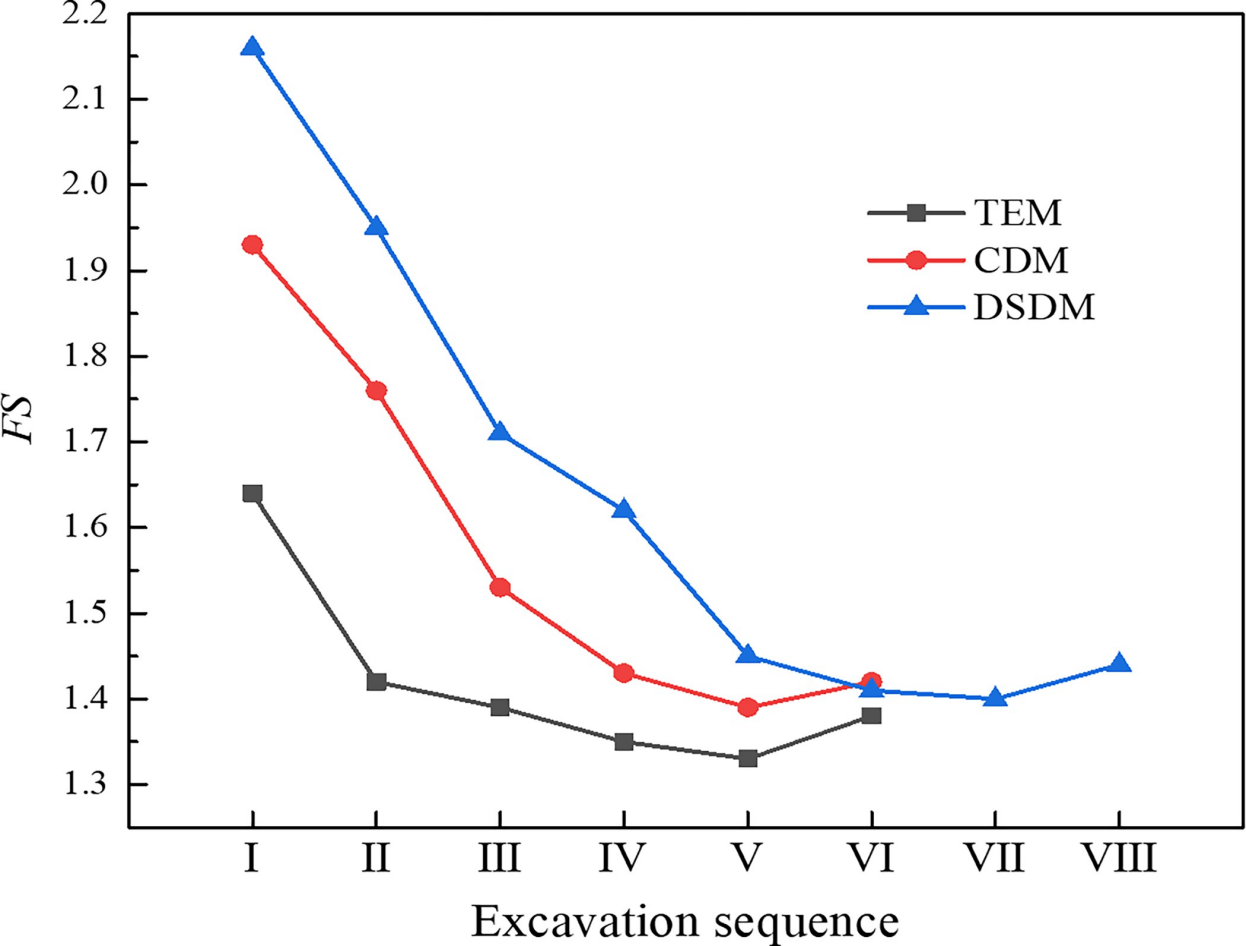
Slope safety factors under various excavation methods.

Fig 8 shows the slope safety factors at tunnel portal under various excavation methods. Before the tunnel portal was excavated, the simulation result of *FS* was 2.54, indicating that the slope was in a stable state. The excavation of the tunnel portal would evidently reduce the slope stability. After the excavation of drift Ⅰ in each method, the value of *FS* was drastically reduced. As the excavation continued, the value of *FS* also continuously reduced. After the secondary lining was implemented, the *FS* increased slightly. The influence of various excavation methods on the slope safety factor was that the smaller the section area in each excavation, the slower the *FS* decreased. In the TEM, after the excavation of bench Ⅴ was completed, the *FS* dropped to 1.33, which was remarkably close to the limit value of 1.30 in the code [35]. The slope was highly likely to be unstable, which had an immense potential safety hazards to construction personnel. In the CDM and DSDM, the *FS* was reduced to the minimum after the temporary support was removed, which dropped to 1.39 and 1.40, respectively. These values met the safety standard, and the slope was in a stable state.

### 4.4 Discussion of simulation results

According to the simulation results, the TEM was not recommended in the excavation of super-large cross-section tunnel portal under a complex stratum. During the excavation by the TEM, due to a large excavation span and a long time for the support to be closed into a ring, the degree of disturbance to the surrounding rock and the range of influence caused by the excavation were evident. The surrounding rock stress was largely released and led to a considerable loose rock load on the tunnel arch. As a result, the surrounding rock deformation was evident, and the concrete at the vault was prone to cracking because of the excessive tensile stress. In addition, affected by the shallow buried depth, forming a bearing arch in the surrounding rock above the tunnel was difficult, and an extensive range of loose areas extended to the surface, which produced noticeable surface subsidence. The CDM and DSDM divided the super-large cross-section into several small drifts and excavated in order. Through span reduction and more timely application of support measures, the area of loose surrounding rock above the tunnel arch could be effectively reduced, and the surface subsidence and surrounding rock deformation could be slowed down. The supporting structure was closed into a ring for a brief time in each drift, which made the stress of concrete more reasonable and safer, so that the bearing capacity of the surrounding rock and the primary support were fully utilized. Compared with the CDM, the DSDM had smaller step-by-step excavation size, shorter excavation and support time of each drift, and fully pre-reinforced the surrounding rock through the construction of the advanced support and temporary support in the leading drift. The mechanical properties of the surrounding rock were effectively improved, which made the self-stabilization capacity of the surrounding rock effective. The excavation and support of multiple small drifts in order could effectively balance the adverse effects of asymmetrical loading at the tunnel portal. Therefore, in the actual tunnel project, the DSDM was adopted to excavate the left tunnel portal.

## 5 Optimization of excavation sequence

**Fig 9:**
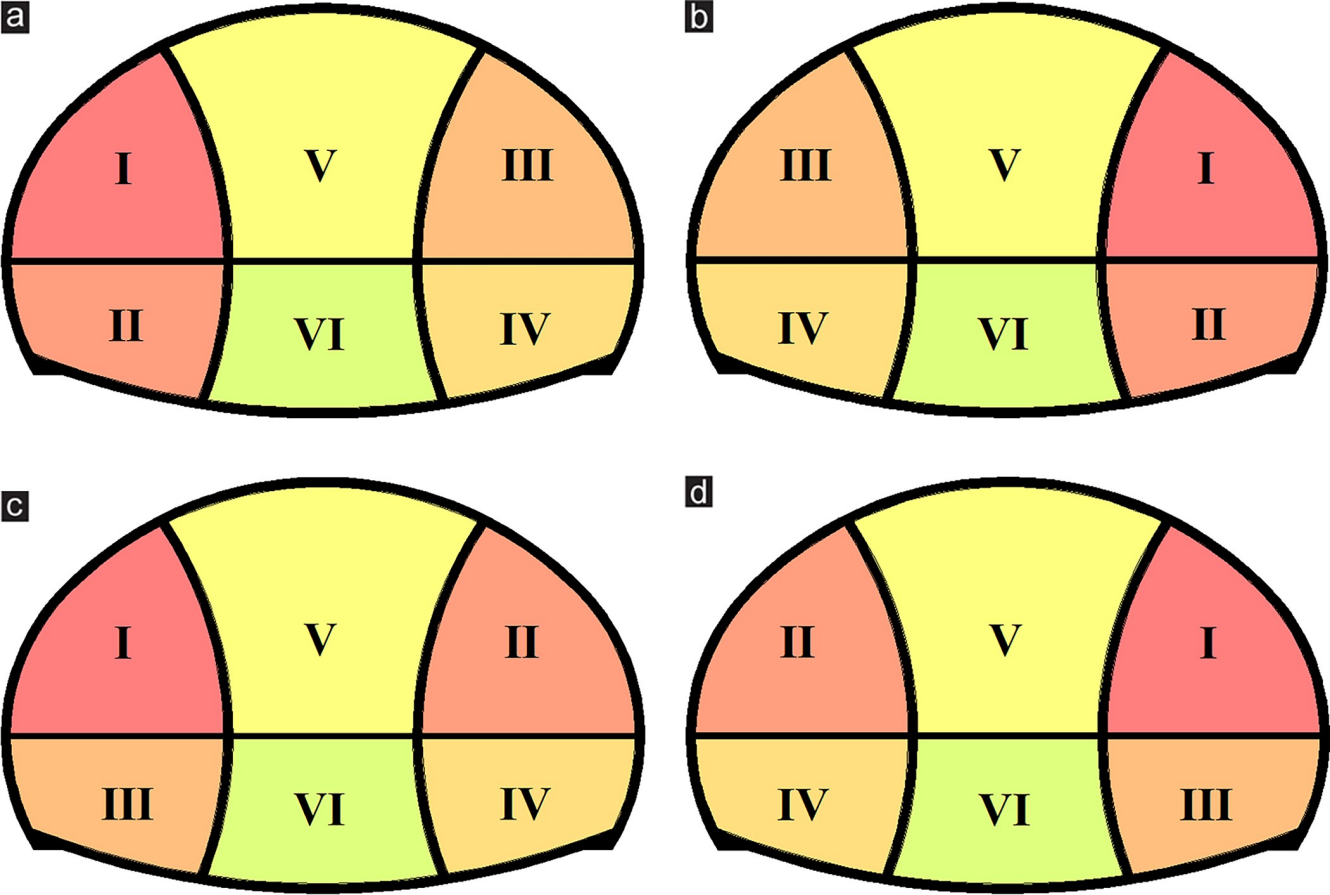
Case conditions of various excavation sequences in DSDM. (a) Case 1, (b) Case 2, (c) Case 3, (d) Case 4.

Previous research results indicated that the excavation sequence in SEM was a key factor affecting the stability of surrounding rock [24]. However, few researchers studied on the excavation sequence of super-large cross-section tunnel portal under shallow buried depth and asymmetrical loading. The excavation sequence must be optimized to find the optimal sequence. In this paper, combining with landform characteristics and existing construction conditions, four excavation sequence cases in the DSDM were proposed, as shown in Fig 9. The excavation sequence of case 1 was as follows: left upper drift Ⅰ at deep buried side—left lower drift Ⅱ —right upper drift Ⅲ at shallow buried side—right lower drift Ⅳ —middle upper drift Ⅴ —middle lower drift Ⅵ. The excavation sequence of case 2 was as follows: right upper drift Ⅰ at shallow buried side—right lower drift Ⅱ —left upper drift Ⅲ at deep buried side—left lower drift Ⅳ —middle upper drift Ⅴ —middle lower drift Ⅵ. The excavation sequence of case 3 was as follows: left upper drift Ⅰ at deep buried side—right upper drift Ⅱ at shallow buried side—left lower drift Ⅲ —right lower drift Ⅳ —middle upper drift Ⅴ —middle lower drift Ⅵ. The excavation sequence of case 4 was as follows: right upper drift Ⅰ at shallow buried side—left upper drift Ⅱ at deep buried side—right lower drift Ⅲ —left lower drift Ⅳ —middle upper drift Ⅴ —middle lower drift Ⅵ.

### 5.1 Comparative analysis of various excavation sequences

**Fig 10:**
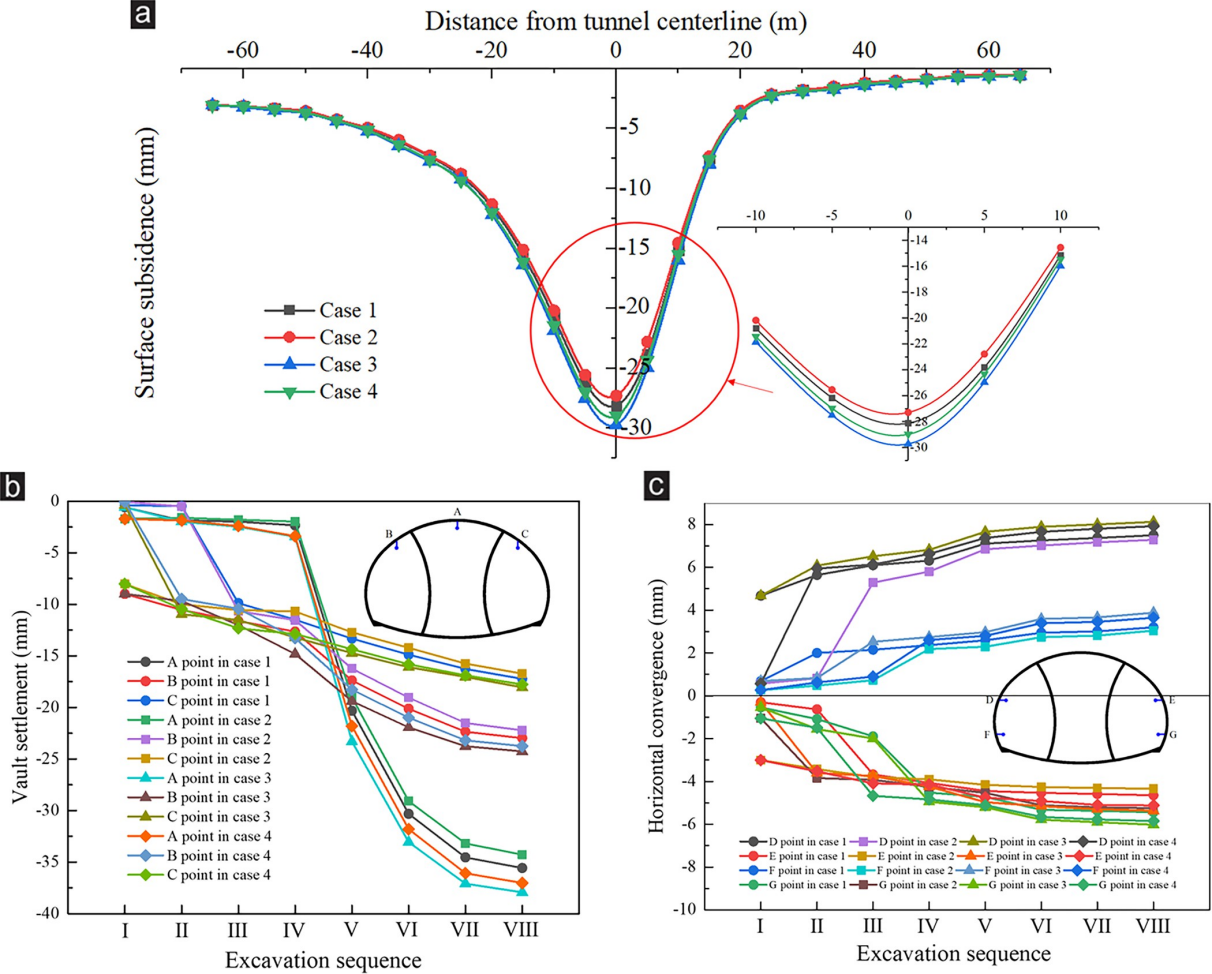
Surrounding rock deformation at ZK119+580 section under various excavation sequences. (a) Surface subsidence, (b) Vault settlement, (c) Horizontal convergence.

Fig 10 presents the simulation results of surrounding rock deformation under various excavation sequences, and Fig 10A shows the surface subsidence. The maximum subsidence in the four cases were 28.12, 27.29, 29.72, and 28.97 mm. The subsidence generated was the smallest in case 2, and the subsidence generated in case 3 or case 4 was slightly greater than that in case 1 or case 2. In excavating the super-large cross-section tunnel, the span reduction effect of excavating one side drift first had a certain positive effect on the control of surface subsidence. Fig 10B and 10C present the vault settlement and horizontal convergence, respectively. The surrounding rock deformation in case 1 or case 2 was less than that in case 3 or case 4, and the deformation of the shallow side drift excavated first (case 2) was slightly smaller than that of the deep side drift excavated first (case 1). Taking the maximum vault settlement as an example (point A), the settlement values under the four cases were 35.58, 34.28, 37.93, and 37.02 mm. Compared with case 3, the maximum vault settlement in case 2 could be reduced by about 9.6%. Therefore, from the perspective of surrounding rock deformation, optimizing the excavation sequence in the project to case 2 was recommended, that is, the excavation should follow the order: shallow buried side drifts > deep buried side drifts > middle drifts.

The stress distribution characteristics of the primary support concrete under various excavation sequences were remarkably similar and displayed only a slight difference in values, as shown in Table 2. The excavation sequence had no evident influence on the stress of the primary support concrete. The concrete stress under the case of excavating one side drift first was slightly better than that under the case of excavating the left and right drift in the upper half bench first. The change of the excavation sequence did not change the stress concentration position on the concrete and had a minimal effect on the stress distribution on the concrete.

**Table 2:** Stress of primary concrete at the ZK119+580 section under various excavation sequences.

Excavation case	Maximum tensile stress/ Distribution location	Maximum compress stress/ Distribution location
Case 1	1.035 MPa/Arch	2.409 MPa/Left arch waist
Case 2	1.029 MPa/Arch	2.433 MPa/Left arch waist
Case 3	1.298 MPa/Arch	2.546 MPa/Left arch waist
Case 4	1.241 MPa/Arch	2.518 MPa/Left arch waist

**Fig 11:**
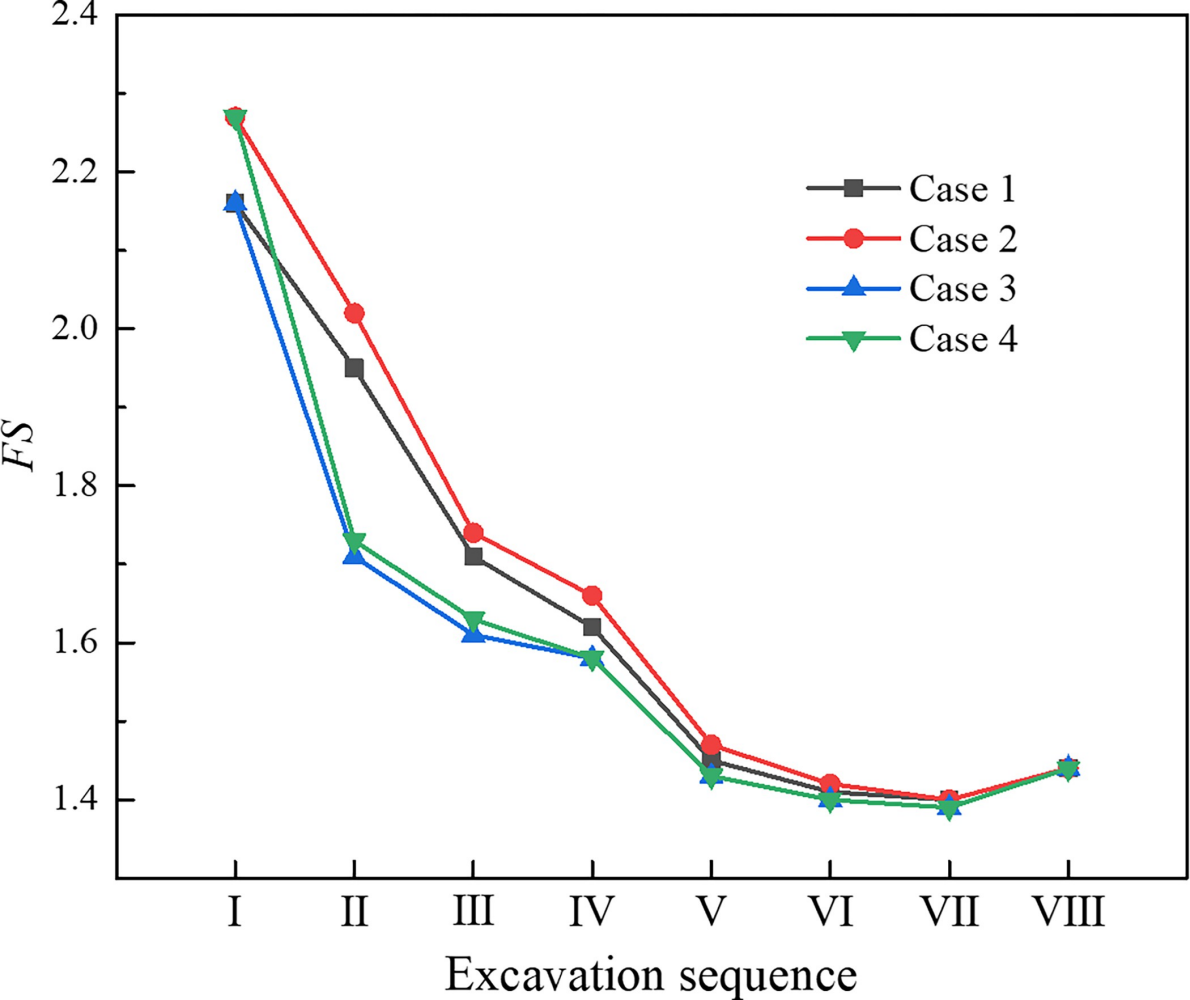
Slope safety factor under various excavation sequences.

Fig 11 presents the safety factor of slope under various excavation sequences. The excavation sequence influenced the slope stability in the early stage of construction. The *FS* in case 1 or case 2 was higher than that in case 3 or case 4 during steps I–V, and the *FS* in case 2 decreased the most slowly. During the early construction stage of the tunnel, the slope under the drifts on one side excavated first was more stable than that the drifts on both sides of the upper half bench excavated first. Among them, excavating the shallow buried side drifts first was more conducive to the slope stability. However, after the middle drifts were excavated (step Ⅵ), the *FS* was reduced to the same in all cases, which meant that the excavation sequence had no influence on the slope stability after the entire tunnel section was formed. Therefore, during the construction of super-large cross-section tunnel portal under shallow buried depth and asymmetrical loading, the slope stability in the early stage of excavation could be improved by changing the excavation sequence of the drift to make the slope transition to the final equilibrium state more smoothly.

### 5.2 Site construction and monitoring

**Fig 12:**
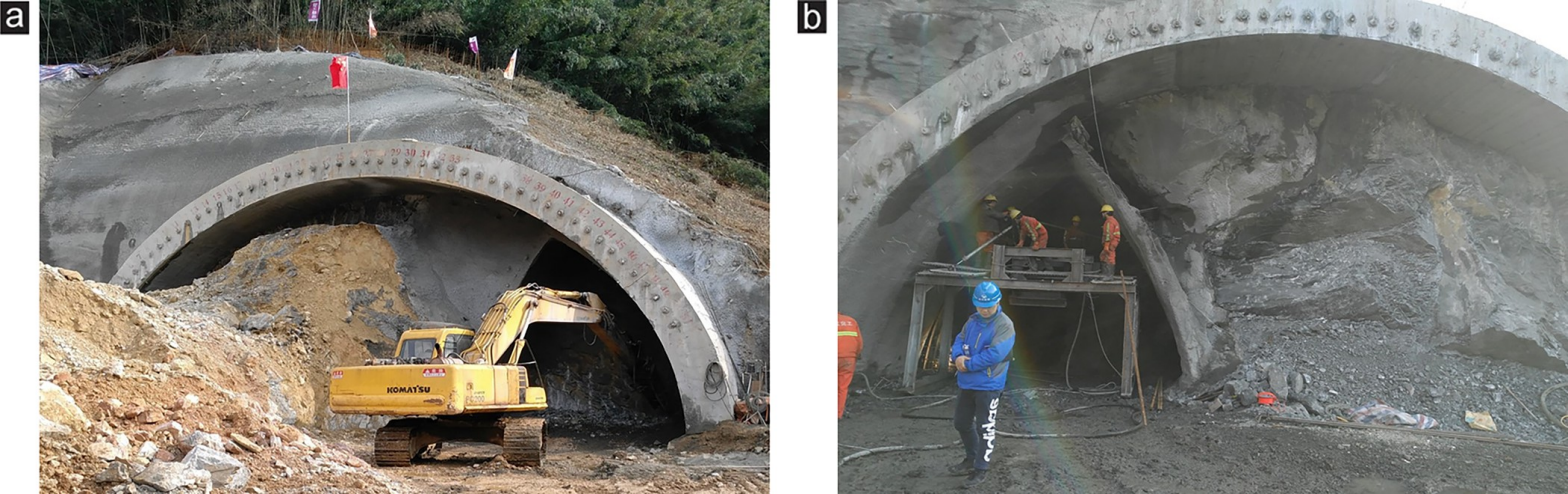
On-site construction at the tunnel portal section. (a) Left tunnel—Case 2, (b) Right tunnel—Case 1.

According to the simulation analysis results, for excavating tunnel portal with super-large cross-section tunnels or extra-large-span tunnels, adopting the DSDM for construction is recommended, whereas the bench methods such as TEM, which are excavation methods without reducing span, are not. In addition, when the tunnel portal is constructed in shallow buried and biased stratum, the excavation sequence of the DSDM can be changed according to the terrain. Excavating the shallow buried side drift pit first and then the deep buried side is recommended. Fig 12 shows the photos of the onsite construction of the tunnel portal by using the DSDM. Fig 12A shows the photo of the left tunnel portal. The drift at the shallow buried side was excavated first after the advanced support was implemented. Fig 12B presents the photo of the right tunnel portal. Because the slope of the right tunnel portal was even on the topography, the effects of case 1 and case 2 were the same during the excavation.

**Fig 13:**
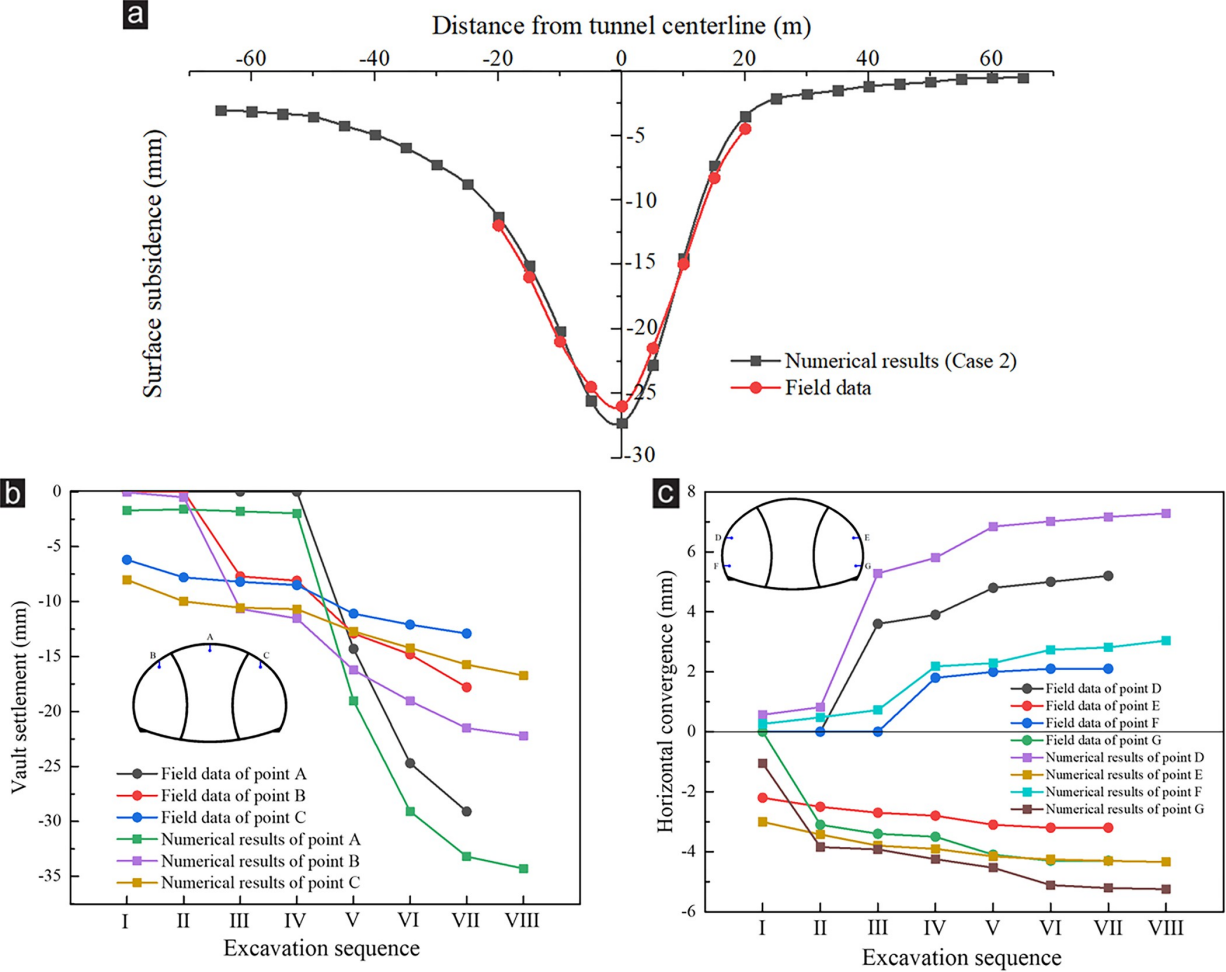
Comparison of surrounding rock deformation between numerical simulation and field monitoring at ZK119+580 section. (a) Surface subsidence, (b) Vault settlement, (c) Horizontal convergence.

Fig 13 compares the surrounding rock deformation between numerical simulation and field monitoring at the ZK119+580 section. The monitoring results of surface subsidence in Fig 13A indicate that due to the influence of the shallow buried and biased stratum at the portal, substantial asymmetrical subsidence appeared on the surface after the tunnel was excavated, and the subsidence at the deep buried side was greater than that at the shallow buried side. The field monitoring data and trend of the surface subsidence were consistent with the simulation results of case 2 in the DSDM. Fig 13B and 13C present the vault settlement and horizontal convergence, respectively. The deformation trend of the surrounding rock obtained by field monitoring was remarkably close to the numerical simulation results. The maximum settlement appeared at the point A (the vault) and reached 29.1 mm, whereas the maximum convergence appeared at the point D (the left arch waist) and reached 5.2 mm. Because the field monitoring data did not include the surrounding rock deformation generated before excavation, the simulation results were greater than the monitoring data. The field monitoring data also present that the surface subsidence and surrounding rock deformation at tunnel portal were effectively controlled after the optimized excavation scheme was adopted. During the actual construction, the slope of the tunnel portal did not crack, and no evident peeling, cracking or water seepage was observed in the primary support or secondary lining in the tunnel. The construction of the tunnel portal was smooth, which indicated that the optimized excavation scheme was reasonable and feasible.

## 6 Conclusions

In this paper, a super-large cross-section tunnel built in shallow buried and biased stratum was taken as an engineering case. The TEM, CDM and DSDM were compared and analyzed from the perspective of controlling surrounding rock deformation, making the support structure stress better, and improving the slope stability. And then the excavation sequence of the DSDM was optimized according to the topographic characteristics of the tunnel portal. The main conclusions are as follows:

1. Due to the influence of the shallow buried depth, asymmetrical loading, and super-large cross-section, the surface subsidence and vault settlement were substantial after tunnel portal excavation. The surrounding rock deformation and primary support concrete stress all exhibited evident asymmetry. The main manifestation was that the deformation or stress at the deep buried side was substantially greater than that at shallow buried side. The maximum surface subsidence appeared directly above the tunnel axis. The slope stability was related to the excavation area. The smaller the section area of the excavation, the slower the reduction of the slope safety factor.
2. The TEM had a large excavation span that resulted in a larger disturbance degree and influence range to the surrounding rock. The loose load acting on the structure was very considerable, which could cause excessive rock deformation and support stress. The CDM and DSDM divided the super-large cross-section into several small blocks and excavated them in an orderly manner. Through span reduction and the timely implementation of temporary supports, the surrounding rock deformation could be effectively controlled, and the structure stress could be better, and the slope stability during early construction could be improved. The excavation area of each drift in the DSDM was smaller, and the support could be closed faster to give full play to the self-supporting capacity of surrounding rock and the bearing capacity of support.
3. The excavation sequence of the DSDM was optimized and analyzed. During tunnel portal excavation under shallow buried depth and asymmetrical loading, excavating the drifts at the shallow buried side first was conducive to controlling the surrounding rock deformation and improving the slope stability in the early stage of construction. The optimized excavation scheme was applied to the actual project. The surface subsidence and surrounding rock deformation obtained by field monitoring were consistent with the simulation results, and the deformation was within the controllable range. During the actual construction, the slope of the tunnel portal did not crack, and no evident peeling, cracking, or water seepage in the concrete was observed, which indicated that the optimized excavation scheme was reasonable and feasible.

## References

[1] Guan B. Collection of design outline on tunnel. Beijing: China Communications Press. 2003. (in Chinese)

[2] The Professional Standards Compilation Group of People’s Republic of China. Code for design of road tunnel (JTG 3370.1–2018). China Communications Press, Beijing. 2019. (in Chinese)

[3] Qi HP, Lu W, Zhang TT, Chen HB, Chi ZM, Xu S, Zhang P. Research on bearing mechanism and spatial layout designing parameters of arch support in large section tunnel. Geotechnical and Geological Engineering. 2019; 37: 4421–4434. doi: 10.1007/s10706-019-00918-w

[4] Wang QB, Xin Z, Jiang B, Sun HZ, Xiao YC, Bian W, Li LN. Comparative experimental study on mechanical mechanism of combined arches in large section tunnels. Tunnelling and Underground Space Technology. 2020; 99: 13386. doi: 10.1016/j.tust.2020.103386

[5] Li ST, Tan ZS, Wu JK, Du WT. Performance of large cross-section tunnel constructed in loose ground by optimal multi-step excavation method. Arabian Journal of Geosciences. 2020; 13(18): 1–8. doi: 10.1007/s12517-020-05961-z

[6] Zhao R. Analysis for bearing performance and construction mechanical behavior of supporting structure for the large cross-section tunnel by half bench CD method. PLoS ONE. 2021; 16(8): e0255511. doi: 10.1371/journal.pone.0255511 34358266 PMC8345873

[7] Salehi B, Golshani A, Rostami J, Schneider-Muntau Barbara. Simulation of complex support systems for large span tunnels: Investigation on support interferences and effects of constitutive models. KSCE Journal of Civil Engineering. 2024; 1–16. doi: 10.1007/s12205-024-5581-4

[8] Sun SQ, Jiang ZB, Li LP, Qiu DC. Model test and numerical verification of surrounding rock stability of super-large-span and variable-section tunnels. Tunnelling and Underground Space Technology. 2024; 153: 106020. doi: 10.1016/j.tust.2024.106020

[9] Ji L, Yao YK, Zhou CB, Zhang Z, Cao HQ, Wu TY. Research on cumulative damage effects and safety criterion of surrounding rock in bench blasting of a large cross-section tunnel. Alexandria Engineering Journal. 2024, 108: 626–639. doi: 10.1016/j.aej.2024.07.099

[10] Zhong ZL, Shen Z, Qiao HY, Li YP, Zhu KX. Study on mechanism of water and mud inrush in deep-buried large-section tunnel crossing water-rich fault fracture zone. Rock Mechanics and Rock Engineering. 2024. doi: 10.1007/s00603-024-04176-y

[11] Jiang Y, Chen R, Shi WH. Analysis of influencing factors in ground subsidence triggered by double-line tunnel excavation under different compressive states. Environment Earth Sciences. 2024, 83: 574. doi: 10.1007/s12665-024-11860-3

[12] Zheng ZJ, Xue XJ, Su D, Chen JF, Qiu T, Chen P, et al. Instability mechanism and reinforcement measures for segments of Ultra-Large diameter shield tunnels when constructing cross passages by mechanical methods. Tunnelling and Underground Space Technology. 2024; 154: 106125. doi: 10.1016/j.tust.2024.106125

[13] Xiao JZ, Dai FC, Wei YQ, Min H, Xu C, Tu XB, et al. Cracking mechanism of secondary lining for a shallow and asymmetrically-loaded tunnel in loose deposits. Tunnelling and Underground Space Technology. 2014; 43: 232–240. doi: 10.1016/j.tust.2014.05.013

[14] Lei MF, Peng LM, Shi CH. Model test to investigate the failure mechanisms and lining stress characteristics of shallow buried tunnels under unsymmetrical loading. Tunnelling and Underground Space Technology. 2015; 46: 64–75. doi: 10.1016/j.tust.2014.11.003

[15] Liu XR, Chen HJ, Liu K, He CM. Model test and stress distribution law of unsymmetrical loading tunnel in bedding rock mass. Arabian Journal of Geosciences. 2017; 10(7): 1–11. doi: 10.1007/s12517-017-2949-5

[16] Zhang JR, Wu J, Yan CW, Gou XM, Ye L, Feng JM. Construction technology of super-large section of highway tunnels with four or more lanes in China. China Journal of Highway and Transport. 2020; 33(1): 14–31. (in Chinese) doi: 10.19721/j.cnki.1001-7372.2020.01.002

[17] Chen H, Lai HP, Huang M, Wang G, Tang Q. Failure mechanism and treatment measures of supporting structures at the portal for a shallow buried and asymmetrically loaded tunnel with small clear-distance. Natural Hazards. 2023; 114: 2283–2310. doi: 10.1007/s11069-022-05471-z

[18] Jiang YJ, Wang XS, Li B, Higashi Y, Taniguchi K, Ishida K. Estimation of reinforcing effects of FRP-PCM method on degraded tunnel linings. Soils and foundations. 2017, 57(3): 327–340. doi: 10.1016/j.sandf.2017.05.002

[19] Zhang YX, Shi YF, Zhao YD, Fu LR, Yang JS. Determining the cause of damages in a multiarch tunnel structure through field investigation and numerical analysis. Journal of Performance of Constructed Facilities. 2017; 31(3): 04016104. doi: 10.1061/(ASCE)CF.1943-5509.0000981

[20] Kaya A, Karaman K, Bulut F. Geotechnical investigations and remediation design for failure of tunnel portal section: a case study in northern Turkey. Journal of Mountain Science. 2017; 14(6): 1140–1160. doi: 10.1007/s11629-016-4267-x

[21] Yang C, Hu ZX, Huang D, Guo F. Failure mechanism of primary support for a shallow and asymmetrically loaded tunnel portal and treatment measures. Journal of Performance of Constructed Facilities. 2020; 34(1): 04019105. doi: 10.1061/(ASCE)CF.1943-5509.0001385

[22] Qiu HZ, Chen XQ, Wu QH, Wang RC, Zhao WY, Qian KJ. Deformation mechanism and collapse treatment of the rock surrounding a shallow tunnel based on on-site monitoring. Journal of Mountain Science. 2020; 17(12): 2897–2914. doi: 10.1007/s11629-020-6026-2

[23] Liu BL, Li B, Zhang L, Huang R, Gao HC, Luo SL, Wang T. Disc-cutter induced rock breakage mechanism for TBM excavation in rock masses with different joint shear strengths. Underground Space. 2024; 19: 119–137. doi: 10.1016/j.undsp.2023.12.006

[24] Sharifzadeh M, Kolivand F, Ghorbani M, Yasrobi S. Design of sequential excavation method for large span urban tunnels in soft ground–Niayesh tunnel. Tunnelling and Underground Space Technology. 2013; 35: 178–188. doi: 10.1016/j.tust.2013.01.002

[25] Li PF, Zhao Y, Zhou XJ. Displacement characteristics of high-speed railway tunnel construction in loess ground by using multi-step excavation method. Tunnelling and Underground Space Technology. 2016; 51: 41–55. doi: 10.1016/j.tust.2015.10.009

[26] Zhao Y, He HW, Li PF. Key techniques for the construction of high-speed railway large-section loess tunnels. Engineering. 2018; 4: 254–159. doi: 10.1016/j.eng.2017.07.003

[27] Luo YB, Chen JX, Shi Z, Li JZ, Liu WW. Mechanical characteristics of primary support of large span loess highway tunnel: a case study in Shaanxi Province, Loess Plateau, NW China primary. Tunnelling and Underground Space Technology. 2020; 104: 103532. doi: 10.1016/j.tust.2020.103532

[28] Xiao JZ, Dai FC, Wei YQ, Xing YC, Cai H, Xu C. Comparative analysis of excavation schemes for a tunnel constructed through loose deposits. Journal of Performance of Constructed Facilities. 2016, 30(4): 04015061. doi: 10.1061/(ASCE)CF.1943-5509.0000813

[29] Li LP, Shang CS, Chu KW, Zhou ZQ, Song SG, Liu ZH, et al. Large-scale geo-mechanical model tests for stability assessment of super-large cross-section tunnel. Tunnelling and Underground Space Technology. 2021; 109: 103756. doi: 10.1016/j.tust.2020.103756

[30] Chen JX, Xu ZL, Luo YB, Song JK, Liu WW, Dong FF. Application of the upper-bench CD method in super large-span and shallow tunnel: a case study of Letuan Tunnel. Advances in Civil Engineering. 2020; Article ID: 8826232. doi: 10.1155/2020/8826232

[31] Cao CY, Shi CH, Lei MF, Peng LM, Bai RX. Deformation characteristics and countermeasures of shallow and large-span tunnel under-crossing the existing highway in soft soil: a case study. KSCE Journal of Civil Engineering. 2018; 22(8): 3170–3181. doi: 10.1007/s12205-017-1586-6

[32] Zhao MJ, Cheng Y, Song ZP, Wang T, Zhang YW, Liu BC. Optimization of construction parameters and deformation characteristics of large-section loess tunnel: a case study from Xi’an metro. Advances in Civil Engineering. 2021; Article ID: 6639089. doi: 10.1155/2021/6639089

[33] Chen H, Lai HP, Qiu YL, Chen R. Reinforcing distressed lining structure of highway tunnel with bonded steel plates: case study. Journal of Performance of Constructed Facilities. 2020; 34(1): 04019082. doi: 10.1061/(ASCE)CF.1943-5509.0001363

[34] Paraskevopoulou C, Diederichs M. Analysis of time-dependent deformation in tunnels using the Convergence-Confinement Method. Tunnelling and Underground Space Technology. 2018; 71: 62–80. doi: 10.1016/j.tust.2017.07.001

[35] Ministry of Housing and Urban-Rural Development of the People’s Republic of China. Technical code for building slope engineering. (GB 50330–2013). Beijing: China Architecture and Building Press. 2013. (in Chinese)

